# Septic Pulmonary Embolism Case Report: Optimal Outcome after Insertion of an Inferior Vena Cava Filter in a Patient with *Staphylococcus aureus* Bacteraemia

**DOI:** 10.1155/2010/651023

**Published:** 2010-06-07

**Authors:** Isabel Esteves, Sofia Vidal Castro, Francisco Abecasis, Cristina Camilo, Marisa Vieira, Dinis da Gama, Manuela Correia

**Affiliations:** ^1^Child and Family Department, Santa Maria Hospital, Intensive Care Unit, Avenida Professor Egas Moniz, 1649-035 Lisbon, Portugal; ^2^Paediatric Intensive Care Unit, Child and Family Department, Santa Maria Hospital, Lisbon Medical Faculty, Lisbon, Portugal; ^3^Vascular Surgery Department, Santa Maria Hospital, Lisbon Medical Faculty, Lisbon, Portugal

## Abstract

A 14-year-old patient presented with bilateral pneumonia and pleural effusions, septic arthritis of the hip, deep venous thrombosis, and pulmonary thromboembolism. Methicillin-sensitive *Staphylococcus aureus* (*S. aureus*) containing the Panton Valentine Leukocidin (PVL) genes was isolated. Contraindication to anticoagulation prompted inferior vena cava filter placement. He completed 4 weeks of treatment with flucloxacillin, with good clinical outcome. *S. aureus* containing PVL genes should be sought in cases of necrotizing pneumonia as it seems to increase the risk of severe multifocal infection and thrombotic complications. There are few reports of placement of filters during *S. aureus* sepsis and bacteraemia. This case highlights that when anticoagulation is not feasible, an inferior vena cava filter can be inserted safely, even in patients with active sepsis and high risk for seeding of the filter. Long-term follow-up confirmed a successful outcome with sterilization of the septic thrombosis with no further pulmonary embolism or additional sepsis episodes.

## 1. Introduction

*Staphylococcus aureus *(*S. aureus*) may cause community-acquired multifocal severe disease on otherwise healthy children and adolescents. Multiple virulence factors have been identified as important to the pathogenesis of infection [1]. One of those factors is Panton-Valentine Leukocidin (PVL), an exotoxin that destroys leukocytes by pore-forming activity. Several studies have shown an association of *S. aureus* containing PVL genes with a more severe inflammatory response [2, 3] and clinically with necrotic skin lesions and abscesses [4], arthritis/osteomyelitis complicated by septic deep venous thrombosis [3, 5] and severe necrotizing pneumonia [6]. However, the role of PVL in the pathogenesis of community-acquired infections, especially in methicillin-resistant strains, is still under discussion [1, 7, 8].

The management of major deep venous thrombosis in septic patients may be difficult. The high risk for complications such as worsening sepsis, metastatic infection, and pulmonary embolism, is an important argument to consider placement of an inferior vena cava (IVC) filter. Those complications also raise questions about the safety and long-term effectiveness of central venous filters, inserted during active sepsis and bacteraemia. Greenfield and Proctor [9] reviewed 175 septic patients who received intravascular filters and found no reports of patients with recurrent sepsis episodes or any significant adverse events. On the other hand, an IVC filter has been shown to become infected during active sepsis [10].

Considering these issues, the authors report a case of safe and effective insertion of an IVC filter in a patient with septic embolism with good long-term outcome.

## 2. Case Report

A 14-year-old male adolescent had a toe trauma complicated with local soft tissue infection and necrosis. Ten days later he presented with fever, cough, thoracic and right inguinal pain. The physical exam revealed signs of minor respiratory distress, 97% oxygen saturation on room air, decreased breath sounds on the right, inflammatory signs of the right lower leg, and a toe wound. Laboratory evaluation at admission showed: leukocyte count 18.2 × 10^9^/L with 91% neutrophils, haemoglobin 1.72 mmol/L, platelets 114 × 10^9^/L, and C-reactive protein 250 mg/L. The chest radiograph was consistent with bilateral pneumonia and minor pleural effusions. The venous doppler ultrasound confirmed a deep venous thrombosis (DVT) of the right femoral vein. Blood cultures were performed and the patient was started on intravenous antibiotic therapy with piperacillin-tazobactam and subcutaneous low-molecular weight heparin. The transthoracic echocardiogram revealed a good ventricular function and no signs of cardiac vegetations.

On the third day after admission, thoracic pain and respiratory distress increased. Considering the hypothesis of septic pulmonary thromboembolism (PT) a chest computed tomography (CT) was performed, showing bilateral thoracic infiltrates and pleural effusion. The diagnostic thoracocentesis revealed a pleural empyema. 

**Figure 1:**
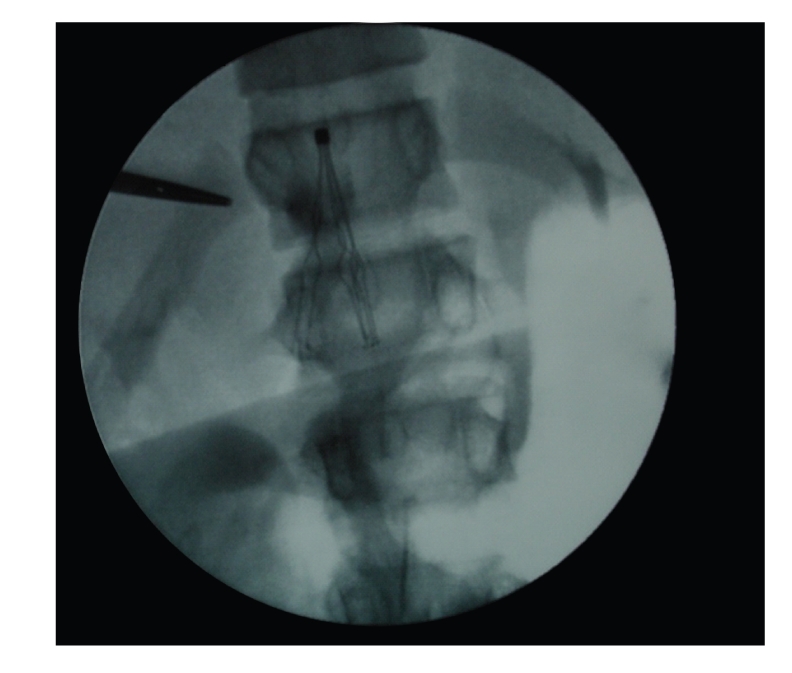
Intraoperative angiography showing inferior vena cava (IVC) filter placement.

Twelve hours later, the patient developed signs of shock and was admitted to the paediatric intensive care unit (PICU). Abdominal ultrasound revealed haemoperitoneum and hepatic hematoma probably due to hepatic puncture during thoracocentesis. Anticoagulation was stopped. A second CT scan showed signs of pulmonary thromboembolism and right hip fluid collection suggestive of septic arthritis. Doppler studies of inferior *vena cava* and ileo-femoral vessels identified a large thrombus in the right iliac veins. The case was discussed with the vascular surgeons and a low-profile percutaneously introducible IVC filter (titanium Greenfield filter, *Medi-tech*, Watertown, MA) was placed (Figure 1). Simultaneously, right hip arthrotomy with debridment and drainage was performed.

**Figure 2:**
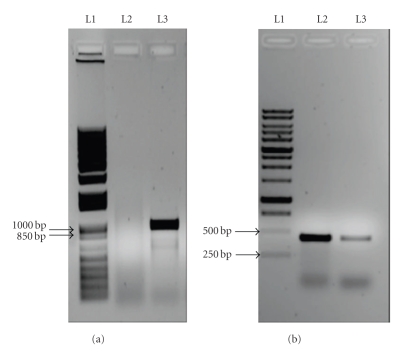
*PCR* amplification of mecA gene products (a) and the genes that code PVL (b) visualized on gel electrophoresis. (a) Lanes (L): L1—mecA gene *PCR* kit; L2—Patient; L3—Positive Control. (b) Lanes (L): L1—PVL gene *PCR* kit; L2—Patient; L3—Positive Control.

Methicillin-sensitive *S. aureus* was isolated from blood cultures and synovial fluid (Figure 2(a)) prompting antibiotic therapy change to flucloxacillin. PVL genes* (lukS-*PV and *lukF*-PV) were detected on *polymerase chain reaction (PCR)* assays (Figure 2(b)). Pleural fluid had no microbiologic isolation.

The patient completed four weeks of intravenous flucloxacillin with good clinical outcome, being afebrile since the sixth day of antibiotic therapy. From a pulmonary standpoint, the patient was stabilized two days after the IVC filter placement, with no other respiratory distress or thoracic pain episodes. Repeated abdominal ultrasounds in the first month after admission showed improvement of haemoperitoneum.

Immunological studies were performed two months after admission, when the patient had completely recovered. Immunodeficiency was ruled out by normal levels of immunoglobulins and phagocyte oxidative capacity (dihydrorhodamine assay). 

The evaluation for venous thrombosis risk factors revealed a common genetic polymorphism of the enzyme methylenetetrahydrofolate reductase (MTHFR) with heterozygosity for 677C>T substitution and another heterozygous polymorphism of plasminogen activator inhibitor 1 (PAI-1) locus with the allelic variant 675G>A (4G/5G), both tested with* PCR* assays. Other diagnostic work-up for hypercoagulability yielded normal results (prothrombin time, activated partial thromboplastin time; antiphospholipid antibodies; levels of fibrinogen, antithrombin III, protein C, protein S; testing for factor V Leiden mutation, and prothrombin G20210A mutation). 

At 14 months of follow-up there were no other episodes of fever or possible bacteraemia, IVC filter migration, progressive IVC thrombosis, pulmonary embolism, or significant hip joint dysfunction.

## 3. Discussion

It is known that *S. aureus* strains harbouring the PVL genes are associated with complicated skin and soft tissue infections [3, 5, 6]. PVL genes are also implicated in bone and joint infections with a higher rate of deep venous thrombosis [2, 5]. These findings are in accordance with the severity of the community-acquired soft tissue infection in our previously healthy patient, complicated with right hip septic arthritis, right femoral venous thrombosis, septic PT, and bilateral pneumonia. In a previous study, the occurrence of septic PT in children infected with a *S. aureus* strain containing PVL genes, represented 19% of the abnormal pulmonary findings in these patients [6]. Deep venous thrombosis should always be considered in patients who present with a musculoskeletal infection and respiratory symptoms, especially if a staphylococcal infection is possible. Also an echocardiography should be routinely performed in this clinical scenario, in spite of conflicting evidence on the importance of isolated *S. aureus* bacteraemia and the risk of endocarditis [6, 11, 12]. 

In the reported case the diagnostic work-up included routine evaluation tests for immunodeficiency and thrombophilia. In spite of the known aggressiveness of *S. aureus* strains harbouring the PVL genes, we believe that hereditary host susceptibility for infection or thrombosis should be sought, especially in young children or whenever there is a family history. Our patient had two common polymorphisms of the MTHFR and PAI-1 genes. Although their clinical significance as risk factors for thromboembolic events is uncertain, they might play a role when associated with *S. aureus* infections.

When septic PT is diagnosed, prompt antimicrobial and anticoagulation therapy should be instituted [5]. In our patient, the anticoagulation had to be stopped because of active bleeding, so a surgical/mechanical solution was sought and an IVC filter was placed.

The significant morbidity and mortality risk of acute disseminated *S. aureus* disease after deep vein thrombophlebitis is well recognized [13]. Remarkably, there are few reports of placement of filters during *S. aureus* sepsis and bacteraemia in children. A retrospective review [14] of 15 children 18 years or younger, suggested that long-standing vena cava filters were safe and prevented pulmonary embolism, but none was placed in the setting of infection. The *Food and Drug Administration* guidance for intravascular filters [15] defends that sepsis and septic embolism are contraindications for IVC filter placement.

An *in vivo* study of the effectiveness of Greenfield filters in animals with infected thrombus, found that antibiotics successfully sterilized the filter, septic embolus, and vena cava wall [16]. On the contrary, different surgical options used in the past, like vena caval ligation, were associated with pockets of infection and abscess, independently of antibiotic therapy [17].

Gonzalez et al. [5] reviewed 3 patients with filter placement after venous thrombosis in *S. aureus* osteomyelitis, and found no filter migration, recurrent septic PT or IVC thrombosis. 

Greenfield and Proctor [9] reviewed 175 septic patients who received intravascular filters and demonstrated in their study that the placement of filters during sepsis did not add to the mortality and was an alternative when anticoagulation was not feasible. The authors found no clinical reports of patients requiring filter removal secondary to sepsis or any other adverse events related to septicaemia following filter placement. The good results may be associated with the inert materials used in the Greenfield filter (stainless steel and titanium). In this way, only a trapped embolus can become infected, a problem that is easily solved with adequate antibiotic therapy. In spite of the significant number of patients included, these results may be biased by the retrospective analysis and lack of specific outcomes measure, with an important number of drop-outs on follow-up evaluation (only 32% of the patients). 

Also, one recent report describes IVC filter insertion in a patient with *Candida glabrata* septic thrombophlebitis that resulted in IVC filter infection [10].

We consider that a severe invasive infection is an important risk factor for venous filter placement, but it seems to be warranted for septic patients who are haemodynamically unstable and cannot tolerate an additional respiratory compromise associated with pulmonary emboli. The indications for this procedure in paediatric sepsis should be reviewed, including significant PT and contraindications for anticoagulation, as in our patient. Risk factors for the development of persistent bacteraemia/fungaemia must also be weighted, such as prolonged intensive care unit stay, other prostetic devices, immunosuppression, and antibiotic resistance. 

This case highlights the importance of considering *S. aureus* strains containing the PVL genes as the etiologic agent when there are signs of bone, joint, or soft tissue infection associated with severe pneumonia and thromboembolic events. In this setting the placement of an IVC filter may be a safe and effective strategy to prevent PT when anticoagulation is not indicated.
